# Alteration of Venous Drainage Route in Idiopathic Normal Pressure Hydrocephalus and Normal Aging

**DOI:** 10.3389/fnagi.2017.00387

**Published:** 2017-11-23

**Authors:** Takeshi Satow, Toshihiko Aso, Sei Nishida, Taro Komuro, Tsukasa Ueno, Naoya Oishi, Yukako Nakagami, Masashi Odagiri, Takayuki Kikuchi, Kazumichi Yoshida, Keita Ueda, Takeharu Kunieda, Toshiya Murai, Susumu Miyamoto, Hidenao Fukuyama

**Affiliations:** ^1^Department of Neurosurgery, Nagahama City Hospital, Nagahama, Japan; ^2^Human Brain Research Center, Kyoto University Graduate School of Medicine, Kyoto, Japan; ^3^Department of Neurosurgery, Kyoto University Graduate School of Medicine, Kyoto, Japan; ^4^Department of Psychiatry, Graduate School of Medicine, Kyoto University, Kyoto, Japan; ^5^Faculty of Health Care Science, Kyoto Tachibana University, Kyoto, Japan; ^6^Department of Neurosurgery, Graduate School of Medicine, Ehime University, Matsuyama, Japan

**Keywords:** normal pressure hydrocephalus, cerebral venous drainage, normal aging, BOLD signal, low-frequency oscillation in systemic circulation, cerebral blood flow

## Abstract

Idiopathic normal pressure hydrocephalus (iNPH) is a highly prevalent condition in the elderly population; however, the underlying pathophysiology in relation to the aging process remains unclear. To investigate the effect of removal of cerebrospinal fluid by lumbar “tap test” on the cerebral circulation in patients with iNPH, 14 patients with “probable” iNPH were studied using a novel blood tracking technique based on blood oxygenation level-dependent (BOLD) magnetic resonance signal intensity. By tracking the propagation of the low-frequency component of the BOLD signal, extended venous drainage times were observed in the periventricular region of the patients, which was reversed by tap test. Interestingly, the venous drainage time in the periventricular region exhibited an age-related prolongation in the healthy control group. Additional regression analyses involving 81 control subjects revealed a dissociation of deep and superficial venous systems with increasing age, presumably reflecting focal inefficiency in the deep system. Our results not only provide insights into the etiology of iNPH, but also point to a potential non-invasive biomarker for screening iNPH.

## Introduction

Syndromes of progressive neurological disturbances, including psychomotor retardation, gait unsteadiness, and urinary incontinence associated with ventricular dilation, in the setting of normal cerebrospinal fluid (CSF) pressure and the absence of papilledema, have been termed as “normal pressure hydrocephalus” (NPH) in Adams et al. (1965). Patients without known precipitating factors are diagnosed with idiopathic NPH (iNPH), the mechanism of which remains largely unknown. However, the steep increase in the incidence of iNPH in individuals who are 60 years of age or older suggests an association with aging (Jaraj et al., 2014; Martín-Láez et al., 2016). Some recent studies have emphasized on the primary role of abnormal water/blood drainage (Williams, 2008; Bateman and Siddique, 2014) or viscoelasticity changes in the brain parenchyma (Owler et al., 2004; Sack et al., 2009) as the likely mechanisms underlying age-related development of the disease (Keong et al., 2016).

Nevertheless, since the initial reports (Hakim and Adams, 1965), the immediate improvement in symptoms following removal of CSF through a lumbar tap has not only been useful for clinical purposes, but has also suggested abnormal perfusion as the direct cause of clinical manifestations (Wikkelsø et al., 1982). Despite the body of evidence demonstrating changes in blood flow following the “tap test” (TT), there are no established diagnostic criteria based on blood flow imaging using single-photon emission computed tomography (SPECT) or positron emission tomography (PET) (Relkin et al., 2005; Keong et al., 2016). It is critical that iNPH be diagnosed sufficiently early to enable CSF diversion using a ventriculoperitoneal or lumboperitoneal shunt (Gallia et al., 2006) where appropriate to prevent irreversible damage. Thus, there is a need for novel, non-invasive techniques to assess this condition in the elderly population.

Recently, mapping the low-frequency phase in a blood oxygenation level-dependent (BOLD) signal time-series has been proposed as a clinically useful biomarker in cerebrovascular diseases (Amemiya et al., 2013; Lv et al., 2013; Christen et al., 2015; Ni et al., 2017). This method is based on a low-frequency oscillation of systemic origin (sLFO), which can be similarly detected using near-infrared spectroscopy from a fingertip and BOLD signals from the brain. There is a constant phase difference across body parts, indicating that this fluctuation of hemoglobin content travels throughout the vasculature (Tong and Frederick, 2012, 2014). The method, termed “lag mapping,” has recently been confirmed to reflect cerebral blood flow (CBF) in general, with high sensitivity to the venous part of the circulation (Tong et al., 2017). Several reports have already indicated that this information can be reliably extracted using conventional functional magnetic resonance imaging settings at a repetition time of 2–3 s, and a duration of 3 min (Anderson et al., 2011; Amemiya et al., 2013). Importantly, because sLFO has its origin in the systemic circulation, this information primarily reflects vascular structure and not neurovascular coupling (Anderson et al., 2011; Taylor Webb et al., 2013; Erdoǧan et al., 2016; Aso et al., 2017a). Moreover, because it is a blood-tracking technique, changes in venous drainage patterns should be reflected in the results. Due to the lack of valves in the cerebral veins, flow direction is known to be reversible across collaterals (Gisolf et al., 2004; Andeweg, 1996).

In the present study, we acquired resting-state BOLD magnetic resonance imaging (MRI) scans before and after a spinal TT, and compared the BOLD lag maps to evaluate the effect of treatment on brain perfusion. Taking advantage of the high sensitivity of the technique in the venous side of the vasculature, changes in drainage time in each part of the brain were measured. Furthermore, we compared the results from patients and healthy control subjects. Our study is the first to apply lag mapping to detect divergence in cerebral blood circulation between the pre- and post-TT states in subjects with iNPH, and to compare it with processes in normal aging, thereby providing new insights into the mechanisms underlying the etiology of iNPH.

## Materials and Methods

### Subjects and Experimental Procedures

The protocol for the present study was approved by the internal ethics review boards of the authors’ hospital and the university. The patients and healthy controls provided informed written consent for the analysis of anonymized MRI scan data and associated clinical data before the scan. All methods were conducted in accordance with approved guidelines from both boards.

**FIGURE 1:**
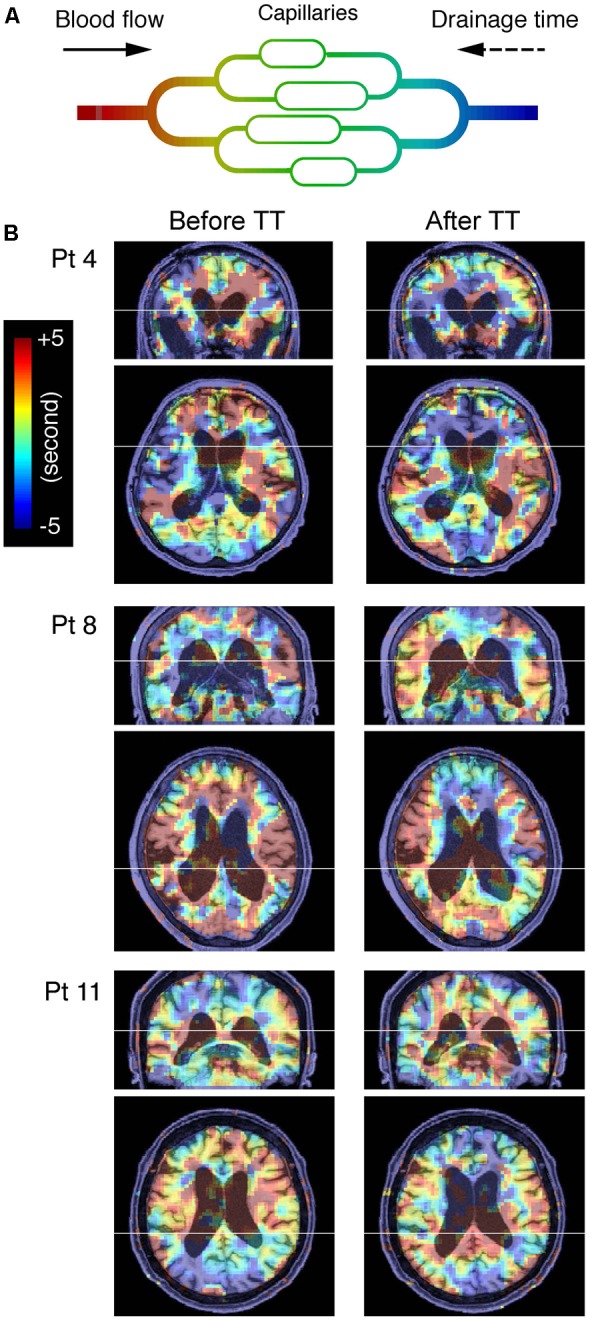
**(A)** Schema of cerebral blood flow and low-frequency oscillation of systemic origin phase. Colors represent the phase tracked by the lag mapping procedure. The phase in each voxel is expressed in the opposite polarity to the convention used in some earlier work, indicating drainage time instead of arrival time. Also note that this “vascular tree” does not directly represent the anatomy of the vascular bed and is expected to change with alterations in the drainage route (Gisolf et al., 2004). **(B)** Blood oxygenation level-dependent lag maps from three representative patients (Pt), before and after the lumbar tap test are overlaid on anatomical images in the original stereotactic space. Warm colors indicate phase advance relative to the global mean signal; these voxels are considered “upstream.” Note that the periventricular regions exhibiting phase shift toward the downstream or venous side of the vasculature are indicated by cool colors.

**FIGURE 2:**
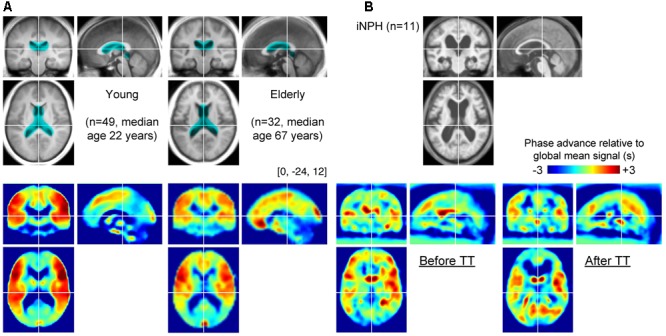
**(A)** Group-averaged T1 anatomical images and lag maps from the control groups. A paradoxical “shrinkage” of the periventricular venous regions with age is noted. The periventricular region of interest (ROI) is overlaid (cyan) on the anatomical images. **(B)** Average lag maps from the idiopathic normal-pressure hydrocephalus (iNPH) patients. The difference between pre- and post-tap test lag maps is eminent in the periventricular region, suggesting recovery of venous drainage to a normal state.

Fourteen patients with “probable” iNPH were consecutively enrolled for evaluation of suspected iNPH symptoms. **Table 1** lists the patients who were finally analyzed (five women; age: range, 76–82 years; median, 81 years). In addition to the set of clinical symptoms, all patients showed ventricular enlargement with a disproportionately enlarged subarachnoid-space hydrocephalus (DESH) pattern on a mid-coronal section of the anatomical image (**Figures 1, 2**). We also ruled out other causes of motor or cognitive symptoms, such as cerebral infarction. Symptoms of iNPH were graded according to the iNPH grading scale (iNPHGS) (Kubo et al., 2008). Two patients (Patients 3 and 4) who exhibited no significant clinical improvement after TT were included in the analysis because of the limited sensitivity of the test (Wikkelsö et al., 1986). Datasets were eliminated from subsequent analyses when lag mapping failed due to excessive motion in both frequency and speed, indicated by a lag histogram with a very narrow peak at zero (see below). As a result, three patients were excluded due to a lack of pre- or post-TT (or both) lag maps, including one of three patients who underwent shunt placement. Of the 11 patients who were analyzed, two patients finally underwent shunt surgery (Patients 4 and 8). One of them (4) did not significantly respond to TT as mentioned above, but the caregiver reported improved communication status. They requested a shunt operation which had a favorable outcome with improved vigilance level and verbal response. Patient 8 with positive TT also underwent shunt surgery with good results.

**Table 1:** Patient demographic information.

Patient	iNPHGC (C/G/U)	MMSE Pre/post-TT	TMT-A (s) Pre/post-TT	TUG (s) Pre/post TT	10 MWT (s) Pre/post TT	General impression of improvement of symptoms after TT
1	1/1/0	27/28	101/48	12.8/12.6	12.0/10.0	+
2	2/1/0	18/21	150/140	8.0/6.9	6.8/8.0	+
3	2/3/2	23/23	115/132	48.9/NT	17.0/NT	–
4^∗^	3/2/3	15/16	NT/NT	23.1/38.6	21.2/30.4	–
5	4/4/4	11/11	375/319	NT/NT	NT/NT	+
6	3/3/3	18/20	183/335	26.5/24.1	31.7/22.3	+
7	4/4/4	12/13	373/308	NT/NT	NT/NT	+
8^∗^	4/3/3	13/14	300/300	63.0/32.6	24.0/17.2	+
9	3/2/3	NT/NT	NT/NT	30.0/23.1	18.5/17.0	+
10	4/3/4	12/14	300/300	26.7/24.3	17.2/16.8	+
11	2/2/1	19/19	123/101	19.7/16.1	19.7/12.7	+

*iNPHGS, idiopathic normal pressure hydrocephalus grading scale; C, cognitive impairment; G, gait disturbance; U, urinary incontinence; TT, tap test; MMSE, mini-mental state examination; TUG, timed up and go test; 10MWT, 10-m walk test; M, male; F, female; +, positive; -, negative. ^∗^Patients who ultimately underwent shunt placement. NT, not testable due to the condition of the patient.*

Control data were obtained from 81 healthy volunteers divided into young (*n* = 49; 18 women; age: range 20–38 years; median, 22 years) and elderly (*n* = 32; 16 women; age: range, 46–81 years; median, 67 years) groups.

### Data Acquisition

A 3 Tesla (T) whole-body MRI system (Skyla, Siemens, Erlangen, Germany) equipped with a 32-channel array coil was used to scan the patients. A 6-min resting-state BOLD acquisition was performed twice each day using the following parameter settings: number of volumes of T2^∗^-weighted, gradient-recalled echo single-shot echo-planar images, 180; field of view = 192 mm; matrix = 64 × 64; 35 oblique axial slices; slice thickness = 3.0 mm; repetition time = 2000 ms; echo time = 30 ms; flip angle = 90°. The cerebellum was only partially covered. Participants were instructed to stay in a supine position in the scanner bore and asked to remain still with their eyes open during BOLD image acquisition. Head restraint pads were inserted to minimize movement. Every patient underwent two experimental sessions: the first, 1 day before the TT; and the second, 1–3 days later.

The control datasets were acquired using a 3T whole-body MRI system (Tim-Trio, Siemens, Erlangen, Germany) equipped with a 32-channel array coil. The acquisition parameters were as follows: field of view = 212 mm; matrix = 64 × 64; 40 oblique axial slices; slice thickness = 3.2 mm with a 25% gap; repetition time = 2500 ms; echo time = 30 ms; and flip angle = 80°. A 10-min scan (242 volumes) was acquired in each experimental session.

For all participants, three-dimensional (3D) T1-weighted images of the brain were acquired for DARTEL in the patients (see below), and spatial normalization unified with tissue segmentation in control subjects (Ashburner and Friston, 2005). A dual-echo gradient echo dataset for B0 field mapping was also acquired before or after the BOLD scan.

### Data Processing

#### Preprocessing and Spatial Normalization

The data were preprocessed using FSL5^1^ and SPM12 (Statistical Parametric Mapping [SPM], Wellcome Department of Cognitive Neurology, London, United Kingdom) on MATLAB (MathWorks, Natick, MA, United States). First, off-resonance geometric distortions in the echo-planar imaging data were corrected using FUGUE within FSL5, using B0 field maps derived from the dual-echo gradient echo dataset. The volumes corresponding to the first 10 s of acquisition were discarded to allow for signal equilibrium and compensate for participants’ adaptation to the scanner noise. After inter-scan slice timing correction, which is of critical importance in lag mapping, head motion was compensated for in two steps: data scrubbing (Power et al., 2012) followed by 3D motion correction. The scrubbing procedure involved searching for abrupt head motion exceeding ±3 mm or ±3° per 0.5 s. The contaminated time points were replaced with linearly interpolated values (Mazaika et al., 2009). Temporal band-pass filtering with a narrow passband (0.008–0.07 Hz) was applied for each voxel before lag mapping to ensure that the phase was uniquely determined within the cross-correlation range of 14 s (see below). The filtered time courses in each voxel were then resampled to a sampling interval of 0.5 s (lag tracking). For the control dataset, spatial normalization was performed by unified segmentation and normalization implemented in SPM12 (Ashburner and Friston, 2005), and the images were resliced to a 4-mm isotropic voxel size before the lag mapping procedure.

To perform voxel-by-voxel group analysis of the patient group, DARTEL (Ashburner, 2007) was used on the T1 anatomical images for accurate co-registration of the affected sulcal/ventricular structures. Non-linear warping to the Montreal Neurological Institute template brain followed despite incomplete registration, especially in the vicinity of the ventricles (**Figure 2B**). After spatial normalization of the anatomical images, BOLD lag maps were normalized using these parameters and re-sliced to 2 mm isotropic voxels. As images from the control group were directly aligned with the Montreal Neurological Institute template, voxel-by-voxel comparisons between patients and controls were not performed.

#### BOLD Lag Mapping

**Figure 1A** is a schematic representation of the lag mapping technique, with the sLFO phase serving as a virtual tracer. Following a previously reported methodology, simple seed-based lag mapping with the global mean signal reference was used (Amemiya et al., 2013; Christen et al., 2015; Aso et al., 2017a; Ni et al., 2017). The lag maps were created by calculating the time shift relative to the reference signal, which yielded the maximum correlation coefficients (i.e., cross-correlation peak) for each brain voxel. This was performed by creating a 3D time × space (voxel) × time-shift array, and by calculating the maxima along the third dimension. The lag map assumed discrete values between -7 s and +7 s at an interval of 0.5 s, in which positive values were assigned to the upstream (i.e., arterial side of the circulation) voxels (Aso et al., 2017a). There was a small number of voxels with a cross-correlogram peak of either -7 or +7 s, and a negative peak correlation coefficient, suggesting ambiguity or failure of tracking. These voxels were uniformly assigned a value of -7; however, the results were essentially identical with or without the treatment. All lag maps were resliced to 2-mm isotropic voxels before evaluation.

### Statistical Analysis

First, a voxel-wise paired *t*-test was performed to determine the effect of TT in iNPH patients. Using SPM12, individual lag-map pairs were smoothed using a Gaussian kernel (full-width at half-maximum [FWHM], 6 mm full width at half maximum for standard paired *t*-testing with a height threshold of *p* < 0.005, uncorrected for multiple comparisons over space. The correction for multiple comparisons was performed using a cluster size threshold of *p* < 0.005 (Poline et al., 1997). Although a liberal height threshold was chosen, considering the exploratory purpose of this analysis and the relatively small sample size, the choice of a conservative threshold in the cluster-size inference was expected to suppress the inflation of type 1 errors (Eklund et al., 2016). The same analysis was repeated excluding Patient 3 who showed no improvement with TT.

An additional region of interest (ROI) analysis was performed for between-group comparisons as the voxel-wise comparison was inadequate due to the poor anatomical co-registration between the groups. The periventricular ROI was created to cover the corresponding regions of the TT-related changes detected in the patients (**Figure 2**, cyan).

Finally, to evaluate alterations in the lag map with age, a voxel-by-voxel regression analysis was performed. All lag maps from the 81 control subjects were entered into one model with two factors of interest: age and sex. A stringent threshold of *p* < 0.05 was set, corrected for the false discovery rate, and further discarded the small clusters (<200 voxels), which approximately corresponded to a cluster-level threshold of *p* < 0.01, corrected for multiple comparisons.

## Results

### Effect of Disease and TT on the Lag Map

**FIGURE 3:**
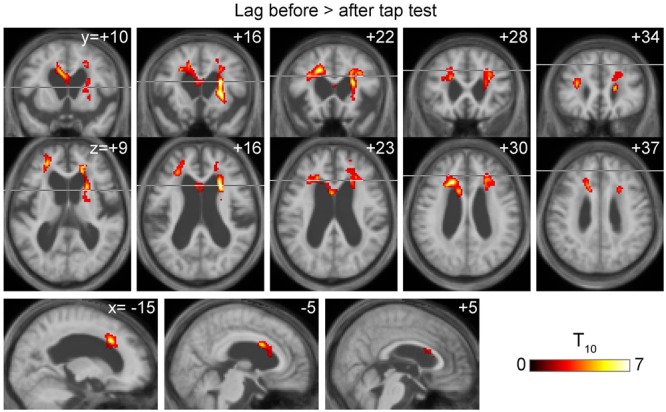
Voxel-wise paired *t*-test revealing a symmetrical set of voxels with significantly shortened drainage time following tap test (TT). It peaked at the vicinity of the ventricular walls of the anterior horns where the internal cerebral vein tributaries run.

The BOLD lag maps from patients with iNPH were highly variable among individuals; however, the changes produced by TT tended to cluster in the periventricular regions (**Figure 1B**). With spatial alignment and averaging among the 11 patients, the difference before and after TT became evident (**Figure 2B**). **Figure 3** illustrates the results of the voxel-wise paired *t*-test to define the effect of TT. Significant shortening of drainage time was observed in two large clusters of voxels around the ventricular walls (*p* < 0.005 in cluster level inference, corrected for multiple comparisons). This cluster coincided with the deep venous system draining into the internal cerebral vein (ICV). The reverse contrast did not yield a significant change at the chosen threshold. An essentially similar result was obtained after excluding Patient 3 with a negative TT, although the cluster was smaller (**Supplementary Figure S1**).

In the control group, some effect of aging was found in the averaged anatomical images and lag maps (**Figure 2A**). In the lag map, red voxels coincide with the middle cerebral artery territories, reflecting early arrival compared with other cortical areas (Takahashi et al., 2014). Similarly, the blue-color areas encompass the periventricular region where the medullary veins drain into the subependymal collecting veins (deep venous system [Beggs, 2013]). However, there was a paradoxical shrinkage of the blue-colored periventricular area in the elderly group, despite their enlarged ventricles, suggesting an age-related change.

**FIGURE 4:**
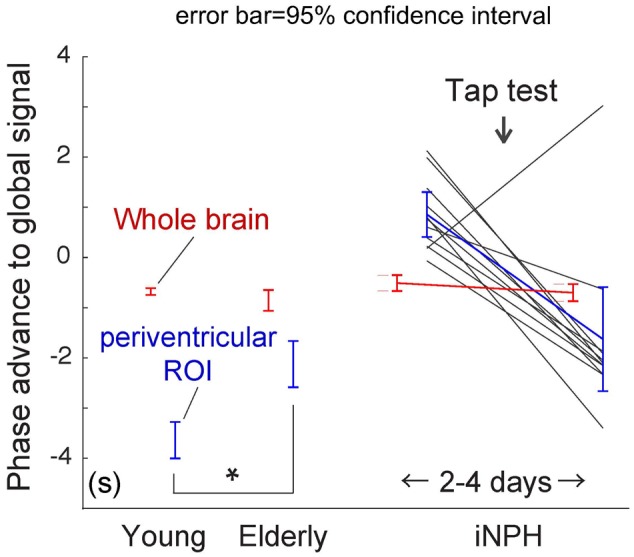
Region of interest (ROI) analysis of the absolute phase advance in seconds. The red and blue lines represent the values from the whole brain and deep venous system ROIs, respectively (the clusters in **Figure 3** are used for the patient group). The values indicate relative drainage time, in that the ROI with the negative phase is in the venous side of the vasculature. The periventricular ROI moves upstream with aging (^∗^*P* < 10^-5^ [two-sample *t*-test]), which is further pronounced in patients with idiopathic normal pressure hydrocephalus (iNPH). After the tap test, the values returned to their normal ranges. The black lines indicate individual patients.

To conduct between-group comparisons, an ROI analysis was used to compensate for deformation in the brains of the patient group (**Figure 4**). The values were extracted from individual lag maps to track the positions of the ROIs in the cerebral vascular tree, and expressed as phase advance (or relative drainage time) to the global signal phase. Following TT, the phase in the SPM clusters (**Figure 3**) shifted downstream by 2.5 s, while the global mean values were invariant to the treatment. Except for one individual (patient 5), the cluster moved to the venous side of the vasculature (relative drainage time <0), indicating that the region was close to the venous outlet of the vascular tree. For the control group, a paraventricular ROI was created to evaluate the regions corresponding to the SPM cluster in iNPH. The results confirmed the short drainage time in the region via ICV in the normal state. However, as suggested by the average maps’ appearance, an age-related effect was found, as reflected by the upstream shift of the ROI (*P* < 10^-5^ [two-sample *t*-test]), implying altered drainage routes in the elderly.

### Effect of Age on the Lag Map

**FIGURE 5:**
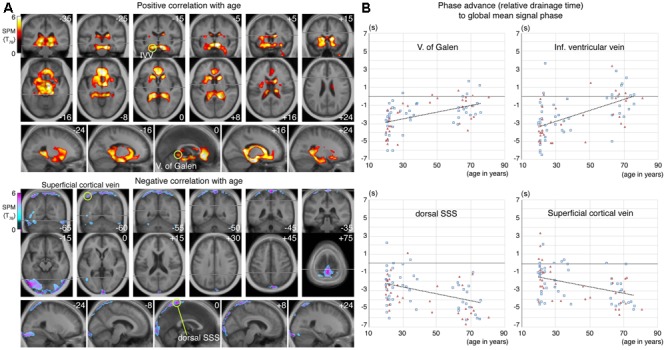
**(A)** Statistical parametric mapping from the voxel-wise regression analysis of the age-related change in lag map. Voxels surviving the threshold of *P* < 0.05, corrected for multiple comparison using the false discovery rate, are shown. An upstream shift of phase (i.e., extended drainage time) is apparent in the regions near the deep venous system, while the opposite was found in the parts of the superior sagittal sinus (SSS) and transverse sinus, with anastomotic veins, all of which belong to the superficial system. Regions of interest (ROI) for four selected local statistical peaks are indicated by green circles. **(B)** Phase values from individual lag maps are plotted for the four ROIs. Subject sex is indicated by different symbols and colors (men: blue rectangles, women: red triangles), to show absence of a strong effect from sex. There is considerable variation in the relative drainage time in all ROIs, suggesting individual variations in the drainage route, but a temporal relationship was found by paired *t*-test between the vein of Galen and the dorsal SSS, only in the elderly group (*P* < 10^-7^).

The effect of aging in the control group was formally investigated using regression analyses. **Figure 5A** illustrate the SPM of positive and negative linear correlations with age, respectively. The regions with extended drainage times according to age were symmetrically distributed along the deep venous system, including the vein of Galen. The cluster extended rostrally to the anterior cingulate cortices along the anterior pericallosal veins, and laterally to the hippocampus along the inferior ventricular veins. In contrast, clusters with shortened drainage time according to age were distributed along the superficial venous system, peaking at the uppermost part of the superior sagittal sinus (SSS).

The lag map phase in each of these regions was extracted for comparison (**Figure 5B**). The local statistical peak in the vein of Galen exhibited an upstream shift by approximately 2 s, while the opposite pattern was observed in the SSS peak. The within-subject phase difference, which should reflect the hierarchical relationship between these two peaks, was not detected in the younger group (*P* = 0.52 [two-tailed paired *t*-test]), but was significant in the elderly group by 3.2 s (*P* < 10^-7^). Such differences were also found between the uppermost SSS and the superficial veins, indicating that the latter is upstream to the former in both the young and elderly groups (0.9 s at *P* = 0.002 in young; and 1.0 s at a marginal significance of *P* = 0.01 in the elderly).

## Discussion

Using a BOLD-based blood-tracking method in subjects with iNPH, we observed an abnormal phase in the periventricular region where the deep veins converge. Under healthy conditions, the phase or relative drainage time in this region consistently exhibited a late venous phase, as reported earlier (Lv et al., 2013; Tong and Frederick, 2014). This abnormally long drainage or “wash-out” time in iNPH was normalized by TT, while the global mean of the phase remained stable. Collectively, these results permit an interpretation that a part of the deep venous system is drained by collaterals in iNPH instead of the normal route via the ICV. The broad change after TT may reflect the normalization of this state, involving a change in the drainage pattern (Andeweg, 1996). Altered venous drainage has been observed in chronic NPH (Kuriyama et al., 2008; Bateman and Siddique, 2014) and the periventricular area may be one of the commonly affected sites of this venous inefficiency.

This interpretation is consistent with a variety of earlier observations. Greitz (1969) reported an increase in ICV size following a shunt operation in three of seven patients, thus suggesting venous collapse before treatment. A similar abnormality in the ICV has also been associated with some types of hydrocephalus (Boller et al., 1970). In relation to hemodynamic dysfunction, Momjian et al. (2004) used PET and reported abnormal CBF with a maximal reduction adjacent to the ventricles. Our results are also in line with those of several SPECT studies demonstrating an increase in CBF after lumbar puncture (Waldemar et al., 1993; Mataró et al., 2003). A recent study investigating the acute effect of TT demonstrated a relationship between clinical improvement and increases in CBF in a similar region using arterial spin labeling (ASL), an alternative, highly reproducible method (Virhammar et al., 2014). Interestingly, Walter et al. (2005) reported a higher time-to-peak than the arrival time following TT in the periventricular area, which was considered unique in this condition. As lag mapping is sensitive to the capillary and venous parts of circulation, combination with ASL may facilitate understanding of these TT-induced changes in different segments of the vasculature. It is also notable that the effect of TT would be merely a partial reversal of the iNPH pathology, which may involve a complex sequence of events (Keong et al., 2016). For example, there should have been a dynamic interplay between the intracranial pressure change by TT and recovery of blood flow, both of which have mutual interaction with impaired brain compliance in iNPH (Eide and Sorteberg, 2010; Goffin et al., 2016). The present finding suggests that the periventricular venous insufficiency, possibly causing local CBF reduction, may be one of the key factors proximal to the clinical manifestation of iNPH (Andeweg, 1996).

The prominent age-related changes in the lag map were unexpected, but may have implications in iNPH pathophysiology, primarily due to the spatial coincidence of the changes. This effect of aging cannot be fully accounted for by cerebral atrophy for two reasons. First, simple dilation of the ventricles in the elderly should result in extension of the periventricular regions, which contradicts the present observation. Erosion of the clusters into the ventricles may be due to magnetic field inhomogeneity in the perivascular CSF space, causing T2^∗^ signal cancelation (Cheng and Haacke, 2001). Second, the dorsal part of the SSS and its tributaries exhibited a completely opposite change, despite the similarly enlarged CSF space due to cortical atrophy. This finding may, therefore, reflect a change in the cerebral venous systems during the normal course of aging.

A short (or negative) drainage time of a region, relative to the sLFO phase of the global mean signal, indicates its position in the venous part of the cerebral vasculature (Aso et al., 2017a). The present results, therefore, suggest that in the elderly, the superficial system moves downstream to serve as the venous outlet because the deep system is not as efficient as it was in the earlier stages of life. Moreover, the superficial clusters in **Figure 5A**, bottom coincide with the location of the anastomotic veins of Trolard, which connect the transverse sinus and the SSS, suggesting involvement of distant collateral pathways. In contrast to the observation in iNPH, this is a new finding and lacks explanation; however, there are lines of evidence regarding age-related stenosis or occlusion of the periventricular vasculature that can be considered (Moody et al., 1995; Farkas et al., 2006).

The fact that both normal aging and abnormalities in iNPH (which is corrected by TT) involve deep venous insufficiency may have etiological implications, as this suggests altered venous drainage in the absence of pathological ventricular dilation. Accordingly, for example, a causal relationship between hydrocephalus and periventricular edema may be questioned (Keong et al., 2016). It can also imply an initiating role of venous congestion in brain compliance reduction which develops during both pathological and aging processes (Eide and Sorteberg, 2010; Beggs, 2013). Although the concept of venous inefficiency as the cause of hydrocephalus is not new (Kuriyama et al., 2008; Williams, 2008), it has not been linked to aging. Although the role of CSF in the mechanism cannot be inferred from the present data, it is interesting that both SPMs in **Figure 5A** encompass regions related to CSF turnover. The lateral lacunae of the SSS, with rich arachnoid granulations, are mainly distributed in the dorsal part, although the role of arachnoid granulations in CSF turnover remains a matter of debate (Grzybowski et al., 2007). Thus, the full picture of underlying mechanisms is yet to be determined for both of these findings in iNPH and normal aging. Nevertheless, the difference in drainage capacity between the superficial and deep systems is of interest because it may possibly create a type of “transmantle pressure” in the brain parenchyma in elderly individuals, which may be a driving force in the disease process.

While we clearly demonstrated a within-subject hierarchical shift in parts of the superficial and deep venous systems, there was, at the same time, a large inter-individual variation in drainage time (**Figure 5B**), suggesting non-uniform drainage pathways. This is consistent with the inter-individual variability of the lag map itself, primarily arising from variations in the vascular structure. Unlike arterial arrival time, imaging of venous drainage time has rarely been performed due to a lack of optimized techniques. Therefore, our findings remain to be confirmed by other methods. It is, nevertheless, a novel advantage of lag mapping that timing can be measured in both directions using the sLFO phase as a virtual tracer directly injected into the brain vasculature (Aso et al., 2017b; Tong et al., 2017). Hence, another clinical implication of the current results is that lag mapping may provide a biomarker for diagnosing, monitoring, and even predicting the development of iNPH. Given the substantial individual variations in the lag map, an ROI analysis or within-individual, annual checkups may be necessary for screening. Our findings warrant a longitudinal study to assess the diagnostic or predictive performance of this novel marker—taking advantage of its non-invasive nature—in the management of this treatable condition in elderly individuals. Limitations of the current study include its relatively small patient cohort and lack of definitive diagnosis of iNPH in most of the cases. Although most subjects experienced clinical improvement after TT, only three underwent ventriculoperitoneal shunting (one of these patients was excluded due to high head movement). In fact, many of our patients and their caregivers finally decided not to undergo the shunt operation due to limited improvements (Iseki et al., 2014). This relatively poor clinical outcome of TT may be due to old age and advanced stage of the disease, both of which are potential sources of bias. Further research involving a larger cohort with a wider range of disease severity should be pursued.

## Ethics Statement

This study was carried out in accordance with the recommendations of Ethics Committee of Kyoto University Graduate School and Faculty of Medicine and Nagahama city hospital with written informed consent from all subjects. All subjects gave written informed consent in accordance with the Declaration of Helsinki. The protocol was approved by both ethics committees.

## Author Contributions

All authors approved the manuscript version to be published. TS and TA contributed to the conception and design of this research, data analysis and interpretation, and drafting of the manuscript. SN contributed to data acquisition, data analysis, and interpretation. TKo, TU, YN, MO, NO, KY, TKi, TKu, KU, TM, and SM were involved in data acquisition and manuscript revision. HF contributed to the conception and design of this research, as well as data interpretation.

## Conflict of Interest Statement

The authors declare that the research was conducted in the absence of any commercial or financial relationships that could be construed as a potential conflict of interest.

## Footnotes

1 http://www.fmrib.ox.ac.uk/fsl

## Supplementary Material

FIGURE S1 SPM result obtained by excluding Patient 3 without clinical improvement after TT.
