# Supplementary material for: Alteration of Venous Drainage Route in Idiopathic Normal Pressure Hydrocephalus and Normal Aging
Source: Front Aging Neurosci. 2017 Nov 23;9:387. doi: 10.3389/fnagi.2017.00387 (PMC5703706; doi:10.3389/fnagi.2017.00387)
Supplement: FIGURE S1 — SPM result obtained by excluding Patient 3 without clinical improvement after TT. [file Image_1.PDF]

Pre>post

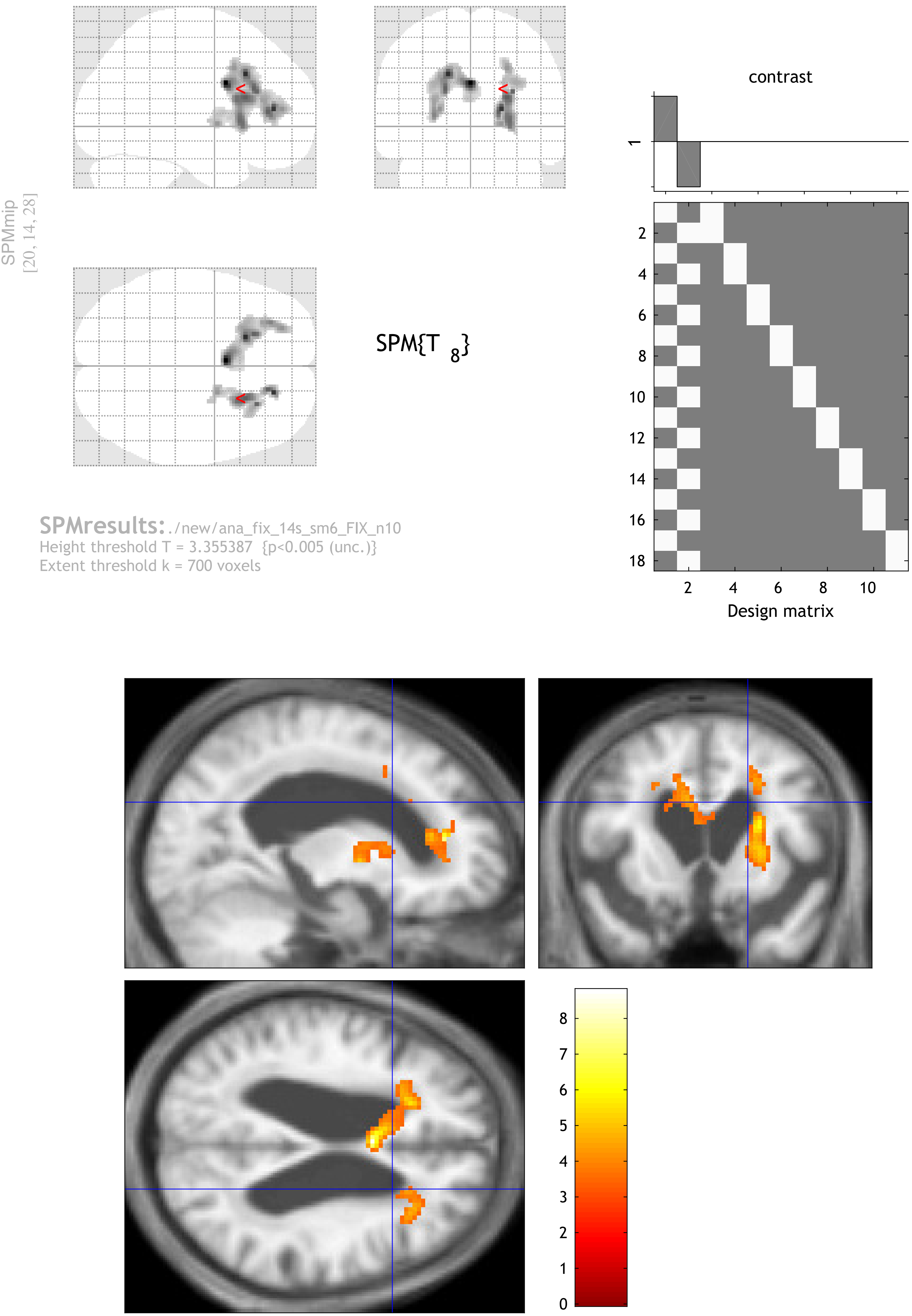
SPMmip
[20, 14, 28]
SPM{T 8}
SPMresults: ./new/ana_fix_14s_sm6_FIX_n10
Height threshold T = 3.355387 {p<0.005 (unc.)}
Extent threshold k = 700 voxels
contrast
1
2
4
6
8
10
12
14
16
18
2
4
6
8
10
Design matrix
8
7
6
5
4
3
2
1
0
